# The Epidemiology and Susceptibility of Candidemia in Jerusalem, Israel

**DOI:** 10.3389/fcimb.2019.00352

**Published:** 2019-10-11

**Authors:** Sarah Israel, Sharon Amit, Ariel Israel, Ayalah Livneh, Ran Nir-Paz, Maya Korem

**Affiliations:** ^1^Department of Internal Medicine, Hadassah-Hebrew University Medical Center, Jerusalem, Israel; ^2^Department of Clinical Microbiology and Infectious Diseases, Hadassah-Hebrew University Medical Center, Jerusalem, Israel; ^3^Clalit Health Services, Jerusalem Research Center, Jerusalem, Israel

**Keywords:** *Candida* species, resistance, antifungal treatment, antifungal susceptibility, epidemiology

## Abstract

**Objectives:** Invasive *Candida* infections pose a major public health problem worldwide and is a major cause of nosocomial bloodstream infection. Our aim was to assess dynamics in incidence, species distribution and antifungal susceptibility of candidemia episodes in Jerusalem, to better understand the epidemiology of invasive isolates and to better direct therapy.

**Methods:** We analyzed the incidence dynamics, species distribution and susceptibility pattern of 899 candidemia episodes during 2005–2016 in Jerusalem.

**Results:** The overall incidence of candidemia was relatively low of 0.62 per 1,000 admissions. *Candida albicans* was the leading pathogen (39.4%); however, there was a shift toward non-*albicans* species, with *Candida glabrata* predominating among them (40%). As expected, more than one-third of candidemias occurred in intensive care units. However, the distribution between species varied and *Candida tropicalis* was the leading pathogen in hematology-oncology patients. The susceptibility of isolates to antifungals remained stable throughout the years. Only a minority of *Candida albicans* isolates were non-susceptible to fluconazole (3.3%), however, an unexpectedly high resistance rate (37.8%) was observed in *Candida parapsilosis* isolates. We found an alarming rate of caspofungin resistance in *Candida glabrata* (33.6%) and *Candida krusei* (67%); this may reflect misclassification of resistance by the *E*-test method.

**Conclusions:** This is the first comprehensive candidemia analysis in the Jerusalem area that should serve as a basis for decision-making regarding appropriate antifungal treatment in the hospital setting. The exceptional high resistance rate amongst *Candida parapsilosis* emphasizes the importance of antifungal susceptibility monitoring in medical centers serving large urban areas to better direct appropriate treatment.

## Introduction

*Candida* species are a frequent cause of nosocomial bloodstream infection (BSI), with an incidence of 0.4–1.5/1,000 admissions and a high mortality rate of 15–49%, despite the availability of potent antifungal agents (Pfaller et al., 2010).

In the recent years, a shift in the distribution of *Candida* species with increased incidence of non-*albicans* species surpassing *Candida albicans* was described (Pfaller et al., 2010). In the SENTRY Antimicrobial Surveillance Program, which monitors global incidence of candidemia and antifungal susceptibilities, non-*albicans Candida* accounted for 52% of candidemia during 2013 with a predominance of *Candida glabrata* and *Candida parapsilosi*s (Castanheira et al., 2016). One of the major issues regarding candidemia is the high geographical variance in the distribution of *Candida* species. For example, in the North America ARTEMIS study, *C. glabrata* which can pose a therapeutic challenge, was the main cause of non-*albicans* candidemia and accounted for 21.1% of cases; whereas in Latin America, *Candida tropicalis* was the leading cause (13.2%) (Pfaller et al., 2010).

Resistance to antifungal agents varies among *Candida* species. Triazole resistance rates among 3,107 *Candida* species were reported in the SENTRY during 2010–2011 as 5.8–13.5% (Pfaller et al., 2013). In 2013, fluconazole non-susceptibility was uncommon among isolates of *C. albicans* and *C. tropicalis* globally, whereas a high rate of fluconazole non-susceptibility was noted among *C. glabrata* and *C. parapsilosis* (20.7 and 19%, respectively) (Castanheira et al., 2016). Resistance to echinocandin was low among all *Candida* species and ranged 0–2.8% (Castanheira et al., 2016). These antifungal susceptibility differences between *Candida* species may lead to different mortality rates. In a study examining fatal cases of candidemia, 61.5, 50, and 41.2% of *C. glabrata, C. parapsilosis* and *C. albicans* isolates, contributed to mortality, respectively (Cheng et al., 2005).

In light of these changes, this study aimed to analyze data of candidemias over a 12-year period in Jerusalem, a large urban area characterized by socially variable population, to estimate incidence and trends in the abundance of various *Candida* species, as well as their resistance patterns. Such data may facilitate decision making regarding the initiation of antifungal drugs and the adequate empiric choice.

## Materials and Methods

### Subjects and Study Design

The study was conducted at Hadassah Medical Center, which is divided into two campuses, one is a tertiary hospital comprising 750 beds and the other is a secondary level hospital of 350 beds. Both hospitals serve a population of 1,000,000 in Jerusalem and the surroundings. Together, the two campuses have 8 main intensive care units (ICUs), 6 internal medicine wards, 4 surgery departments and 3 hematology-oncology departments including bone marrow transplantation. All patients admitted to the hospital who developed candidemia from January 2005 to December 2016 were included in the study. The first episode of candidemia was included for each patient, unless there was more than one episode occurring at least 30 days apart. *Candida* blood isolates from cultures taken 48 h following the admission day were considered nosocomial. Microbiologic data that included identification and antifungal susceptibilities of *Candida* species were retrieved from the electronic information system of the clinical microbiology laboratory. Demographic and clinical data were collected retrospectively from patient electronic medical records. The study was approved by the local IRB.

### Species Identification and Antifungal Susceptibility Testing

During 2005–2012, *Candida* species were identified from BSI using CHROMagar Candida (HiLabs, Israel) and API ID 32 C (bioMerieux, France), therefore up to third of the rare isolates identified by API ID 32 C may have been misidentified (Cendejas-Bueno et al., 2010). From 2012 and on, isolates were identified mainly by matrix-assisted laser desorption ionization time of flight mass spectrometry (MALDi TOF-MS, VITEK MS, bioMerieux, France). Susceptibility testing was performed as part of routine patient care, using the *E*-test method according to manufacturer's instructions (bioMerieux, France). *Candida* isolates were tested for fluconazole, voriconazole, caspofungin, and amphotericin susceptibilities.

Susceptibility results were interpreted according to the Clinical and Laboratory Standards Institute (CLSI) M60 breakpoints (CLSI, 2017). The MIC endpoints of the *E*-test were elevated to the next 2-fold dilution concentration, which matched the dilution schema of the microdilution method. *Candida krusei* was considered universally resistant to fluconazole. In cases in which no clinical or efficacy data were available, we used the proposed epidemiologic cutoff value (ECV) criteria, according to the CLSI M59 document (CLSI, 2018). For the purpose of analyses, we defined resistant isolates as those that were non-susceptible by CLSI breakpoints (MIC > susceptibility CBP), or non wild-type (WT) when ECV criteria were used (MIC > ECV). In cases of *Candida glabrata*, isolates with fluconazole MIC >32 μg/ml were considered resistant. All *Candida* species and antifungal susceptibilities in this study are available as supplementary data (Supplementary Table 1). Interpretive breakpoints and ECVs used in this study are available in Supplementary Table 2.

### Antifungals Consumption Data Analysis

From January 2007 through December 2016, data on the hospital use of fluconazole and echinocandins were obtained from computerized hospital databases. Data about echinocandin use was available from 2011. Consumption was expressed as daily defined doses (DDDs) and normalized per 1,000 patient-days. One DDD is the standard adult dose of an antimicrobial agent for 1 day treatment, defined by the WHO.

### Statistics

All incidence rates were calculated using the summed numbers of inpatient days at Hadassah Medical Center during the study period as the denominator, and are presented per 1,000 inpatient days. Categorical variables were analyzed using the Fisher exact test and continuous variables using the Mann–Whitney *U*-test. We evaluated a possible trend for increase in resistance using a logistic regression model of resistance according to year. All analyses were performed using the R software (version 3.3.3).

## Results

### Epidemiology

Eight hundred ninety-nine candidemia episodes of 889 unique patients were analyzed. Ten patients had two episodes separated by at least 30 days; therefore, each episode was considered independently for analysis. In 20 episodes (2.2%), more than one candida species was identified (poly-candidemia), consequently the analysis included 919 *Candida* isolates. Ninety-seven percent (*n* = 872) of the episodes were nosocomial. During the study period, the annual rate of candidemia did not change markedly and the incidence was stable, with an overall 0.62 episodes per 1,000 hospital admissions. The male to female ratio was 1.7. The adult to pediatric candidemia ratio was 2:1, concordant with the hospital age admission ratio.

During the study period, *C. albicans* was the predominant pathogen (*n* = 368, 39.4%), followed by *C. glabrata* (*n* = 176, 18.8%), *C. tropicalis* (*n* = 168, 18 %), *C. parapsilosis* (*n* = 135, 14.4%), *C. krusei* (*n* = 54, 5.7%), and other *Candida* species (*n* = 32, 3.4%). *C. albicans* was the most common species isolated in adults (34.5%), followed by *C. glabrata* (22.4%); whereas in children, *C. albicans* was isolated in 50%, followed by *C. parapsilosis* (17.7%). *Candida auris* was not isolated.

**Figure 1:**
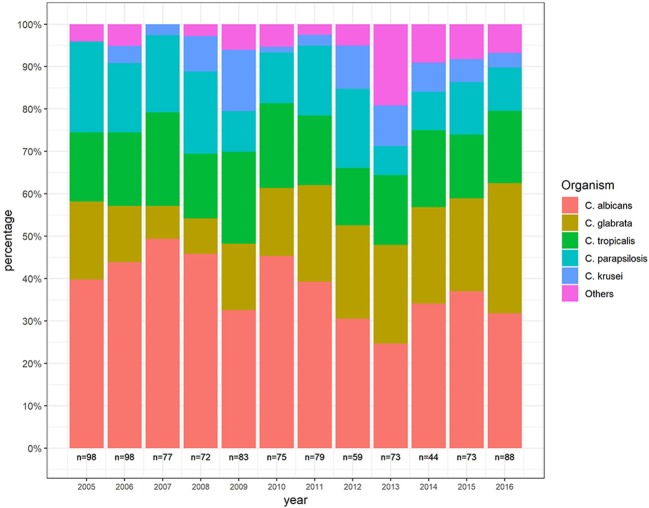
Distribution of *Candida* species during the study period.

Over the years, the proportion of *C. albicans* decreased from 39.6 to 33.7% and the proportion of *C. glabrata* increased from 17.7 to 28.9%, representing about 40% of non-*albicans* species. The proportion of *C. parapsilosis* decreased over the years. Similar trends were not demonstrated in other species (Figure 1).

**Figure 2:**
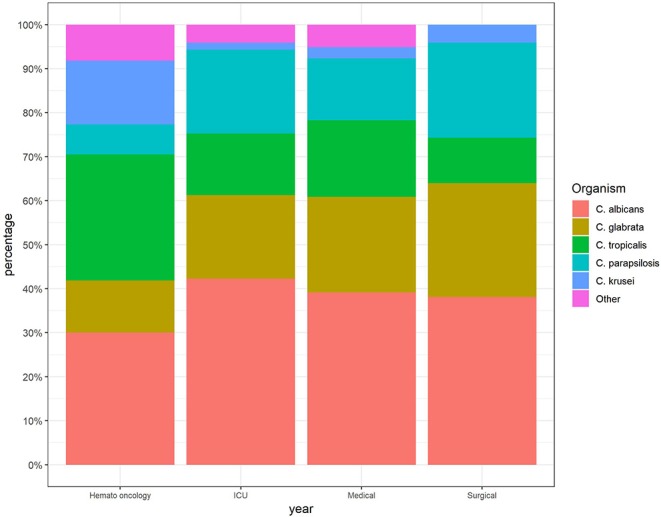
Distribution of *Candida* species according to hospital wards.

The incidence of candidemia was the highest in ICUs and hemato-oncology wards (5.8 and 5 per 1,000 admissions, respectively), whereas in internal medicine and surgery wards, the incidence was low and stable over the study period (1.3 and 0.4 per 1,000 admissions, respectively. The proportion of *C. glabrata* isolates was higher in patients hospitalized in the internal medicine and surgery wards, while in the hemato-oncology wards, the rate of *C. tropicalis* was higher (Figure 2).

### Antifungal Susceptibility Patterns (Figure 3)

**Figure 3:**
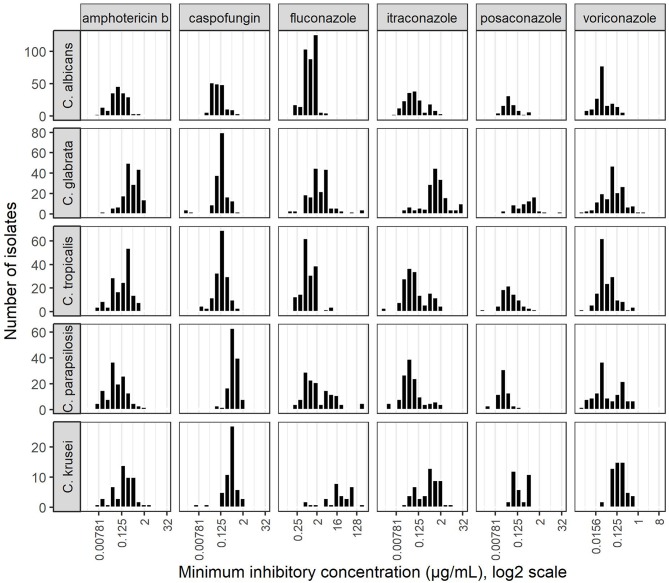
Minimal inhibitory concentration distributions of antifungals against *Candida* spp.

Among the three main groups of antifungals, polyenes, represented by amphotericin B, had the highest susceptibility rates (99.9% of *Candida* species examined were WT according to the CLSI published ECVs). Most *C. albicans, C. tropicalis* and *C. krusei* isolates were triazole susceptible (excluding fluconazole in *C. krusei* cases), or fluconazole SDD (MIC ≤ 32 μg/ml) in *C. glabrata* cases. Resistance rates for fluconazole and voriconazole among *C. parapsilosis* isolates were unexpectedly high, 37.8 and 28.2%, respectively (Table 1) with a bimodal MIC distribution (Figure 3).

**Table 1:** Resistance to antifungal therapy during the study period.

**Candida species^a^**	**Antifungal treatment**			
	**Fluconazole**	**Voriconazole**	**Caspofungin**	**Amphotericin B**
*Candida albicans*	12/368 (3.3%)	4/185 (2.2%)	14/183 (7.6%)	0/184 (0%)
*Candida glabrata*	7/176 (4%)	8/171 (4.7%)	56/168 (33.6%)	0/168 (0%)
*Candida tropicalis*	8/168 (4.8%)	13/165 (8%)	12/164 (7.4%)	0/166 (0%)
*Candida parapsilosis*	51/135 (37.8%)	38/134 (28.2%)	0/133 (0%)	0/135 (0%)
*Candida krusei*	NA	2/54 (3.8%)	36/54 (67%)	1/53 (1.9%)
Overall	78/847 (9.2%)	65/709 (9.2%)	118/702 (16.8%)	1/706 (0.1%)

a *The total number of Candida isolates analyzed in 899 candidemia episodes was 919. The table includes 901 isolates for which there were defined clinical breakpoints or epidemiological cutoff values. Other 18 Candida spp. not included in the table were Candida dubliniensis (n = 7), Candida guilliermondii (n = 2), Candida lusitaniae (n = 2), Candida kefyr (n = 1), Candida ciferrii (n = 1), Candida norvegensis (n = 1), Candida pelliculosa (n = 2), Candida rugosa (n = 1), Candida utilis (n = 1). The MIC range of these species was 0.125–>256 μg/ml to fluconazole, 0.004–0.5 μg/ml to voriconazole, 0.004–0.5 μg/ml to caspofungin and 0.008–>32 μg/ml to amphotericin B*.

Within the echinocandins, most *C. albicans, C. tropicalis* and *C. parapsilosis* isolates were caspofungin susceptible (resistance rates ranging 0–7.6%). We found high caspofungin resistance rates among *C.glabrata* and *C. krusei* isolates, 33.6 and 67.0%, respectively (Table 1).

Triazole resistance did not change significantly among *C. albicans* and *C. tropicalis* isolates since 2005 (*p*-values 0.69, 0.46 for fluconazole, and 0.88, 0.16 for voriconazole; respectively). One third of *C. parapsilosis* isolates were resistant to fluconazole in 2015 (3/9), and half in 2016 (4/8), yet this trend was not statistically significant due to low sample numbers (*p* = 0.71). Likewise, there was no increase in caspofungin resistance over the study period (*p* = 1).

As for drug usage, fluconazole had a stable demand of DDD/1,000 patient-days during 2011–2016 (range 23.3–29.8, mean 26.3, median 26.3). Conversely, an increased use of echinocandins through 2011–2016 was noticed due to higher demands from the ICUs and hemato-oncology wards (range 0.4–1.18, mean 0.98, median 1.18).

## Discussion

Given the high geographical variance in the distribution and resistance patterns of *Candida* species, in this study we analyzed candidemia data over 12 years in a large tertiary center in Israel to better understand contemporary trends that might affect treatment decision making. We found a stable incidence of candidemia (0.62 episodes per 1,000 hospital admissions) and increased rate of non-*albicans* candidemia, mainly attributed to *C. glabrata* accounting for 40% of the non-albicans cases as similar to other reports (Pfaller et al., 2010, 2019). Species distribution varied according to age group and admitting ward. Fluconazole consumption over the study period was stable, concordant with low level resistance rate to fluconazole among *Candida* species totally. Specifically, *C. parapsilosis* isolates had a high rate of resistance to fluconazole as well as voriconazole (37.8, 28.2%, respectively). Increasing echinocandin consumption was not translated to a general increase of resistance to caspofungin, though a high rate of resistance was noted amongst *C. glabrata* and *C. krusei* isolates (33.6 and 67.0%, respectively).

### Species Distribution

Similar to our findings, an increased rate of *C. glabrata* candidemia during the last decade was documented in another tertiary center covering the central district of israel (Eliakim-Raz et al., 2016). Former studies linked the increased rate of *C. glabrata* candidemia to increased fluconazole use (Lortholary et al., 2011). However, high-risk patients in our database did not receive fluconazole prophylaxis, consistent with hospital policy, and there was no increase of fluconazole use from 2007 to 2016. Another study (Ben-Ami et al., 2012) found that recent exposure to antibacterial drugs increased the risk of BSI with fluconazole-resistant *Candida* isolates, suggesting that the effects of antibacterial drugs may have significant impact on azole resistance in *Candida* species. This may explain the increased incidence of *C. glabrata* in our study (Figure 1).

Our analysis also points to a difference in species distribution according to the admitting ward and age group, that can potentially impact empiric treatment choices: As opposed to the general shift toward *C. glabrata*, the main cause to non-*albicans* candidemia in the hemato-oncology wards was *C. tropicalis* (28.6% overall), a generally fluconazole susceptible isolate. *C. tropicalis* originates from the gastrointestinal (GI) tract and has been described in the past as a common cause of candidemia in neutropenic patients (Wingard et al., 1979). Species distribution according to age group underscored *C. parapsilosis* as the main cause of non-*albicans* candidemia in pediatric patients which may reflect increased use of central lines in this population and mandates appropriate empiric treatment considering the high resistance rate to azoles found in this study among these isolates (Trofa et al., 2008).

### Antifungal Susceptibility

The susceptibility analysis in this study was based on *E*-test, an agar-based method widely used in many laboratories for its simplicity. *E*-test has high categorical and essential agreement with the recommended CLSI broth microdilution method (BMD) (Matar et al., 2003).

The low susceptibility rate to fluconazole and voriconazole (62.2 and 71.8%, respectively) among *C. parapsilosis* in this study is meaningful as this is a common non-*albicans* species with increasing prevalence (Pfaller et al., 2019) that may be nosocomially transmitted by hand carriage and cause catheter-related candidemia (Van Asbeck et al., 2007). Generally, fluconazole is considered a highly effective drug against this pathogen, with resistance rates of roughly 3.9% (Pfaller et al., 2019); however, some investigators have reported a rise in the incidence of invasive infections due to fluconazole-resistant strains (Pfaller et al., 2014). This rise is worrisome due to the potential of nosocomial horizontal transmission of resistant strains (Trofa et al., 2008). A national Israeli candidemia study (Ben-Ami et al., 2012) and a recent study from a different district of Israel (Eliakim-Raz et al., 2016), have found markedly lower fluconazole resistance rate among *C. parapsilosis* as compared to ours (13.3 and 11.1% respectively). These local differences reflect the limitations of national based monitoring of *Candida* spp. distribution and antifungal susceptibility profile that can miss such variances and outline an unoptimized therapy, even in a small country as Israel.

We hypothesize that the decreased fluconazole susceptibility shown here, alongside with the bimodal MIC distribution of fluconazole and voriconazole as shown in Figure 3, and a stable demand of fluconazole over the years, reflect the presence of regional resistant clones. These clones may harbor mutations in *ERG11* or overexpress *ERG11*, a gene encoding lanosterol 14α-demethylase which is the target enzyme for fluconazole (Souza et al., 2015). Clonality among *C. parapsilosis* was mainly investigated in outbreak settings in other studies. In an outbreak of 28 *C. parapsilosis* BSI in an ICU in Brazil, typing of 21 fluconazole resistant strains revealed 9 isolates that were considered genetically related with only a minority of patients previously exposed to fluconazole. This cluster suggested the horizontal transmission of fluconazole resistant *C. parapsilosis* (Pinhati et al., 2016). A study that analyzed resistant *C. parapsilosis* by microsatellite typing, confirmed a clonal origin of the strains with all strains harboring a common mutation leading to a Y132F amino-acid substitution in the *ERG11* gene (Souza et al., 2015). Similar to the Brazilian outbreak, the same underlying genomic mechanism may be responsible for our findings and further study examining the possibility of clonality and molecular resistance patterns is currently underway.

Altogether, we did not find an increase in caspofungin resistance among *Candida* isolates. This is in concordance with other studies from the last decade (Posteraro et al., 2015). Nonetheless, the increased echinocandin use during the last 4 years of the study, specifically in hemato-oncology wards, necessitates continuous monitoring of the echinocandin resistance rate.

We suspect that the low proportion of caspofungin susceptibility among *C. glabrata* and *C. krusei* (66.4 and 33%, respectively) is due to the low reliability of the caspofungin *E*-test for these two species. The revised CLSI recommendations refer to a risk of misclassifying caspofungin susceptible *C. glabrata* and *C. krusei* as resistant when tested by the *E*-test, compared to the BMD method (Pfaller et al., 2012). In addition, a study of a large set of *C. glabrata* isolates in Israel (Ben-Ami et al., 2014) found low essential agreement (47.8%) between BMD and *E*-test when caspofungin was tested according to an ECV of 0.12 μg/mL (Pfaller et al., 2011), which is the current susceptibility breakpoint (CLSI, 2017). This breakpoint was found to be unreliable to discriminate between resistant and susceptible isolates because the MIC distribution curve differed from that of the CLSI, and clustered between 0.25 and 0.5 μg/mL, similar to our isolates that also clustered around these values (Figure 3). Therefore, *C. glabrata* susceptibility results, as determined by the *E*-test method for caspofungin should be interpreted with caution; and the validation of susceptibility results with BMD may be advisable for invasive strains (Ben-Ami et al., 2014).

## Conclusions

We observed an increasing proportion of non-*albicans* BSI, similar to other study groups worldwide, with varied species distribution according to admitting ward and age; however, antifungal resistance did not significantly increase over the years. We found a high rate of fluconazole and voriconazole non-susceptibility among *C. parapsilosis* isolates. This may represent a local clonal phenomenon and highlights the importance of prudent selection of empiric therapy. Our reported high rate of caspofungin resistance among *C. glabrata* and *krusei* may reflect misclassification of resistance by the *E*-test. Ongoing surveillance of the epidemiology and antifungal resistance of *Candida* isolates in different geographic regions as well as large urban areas is imperative as it enables tracking clonal patterns with concordant resistance mechanisms; this promotes better selection of empiric antifungal therapy.

## Data Availability Statement

The datasets generated for this study are available on request to the corresponding author.

## Author Contributions

SI: acquisition of data and analysis, drafting of article and critical revision. SA: acquisition of laboratory data, drafting of article and critical revision. AI: analysis of data. AL: acquisition of data and analysis. RN-P: drafting of article and critical revision. MK: conception and design of study, acquisition and analysis of data, drafting of article and critical revision, final approval of manuscript.

### Conflict of Interest

The authors declare that the research was conducted in the absence of any commercial or financial relationships that could be construed as a potential conflict of interest.
