# Supplementary material for: The Epidemiology and Susceptibility of Candidemia in Jerusalem, Israel
Source: Front Cell Infect Microbiol. 2019 Oct 11;9:352. doi: 10.3389/fcimb.2019.00352 (PMC6801307; doi:10.3389/fcimb.2019.00352)
Supplement: Supplementary file 2 [file Table_2.DOCX]

**Supplementary table 2.** CLSI interpretive criteria and epidemiological cutoff values for *Candida* species drug susceptibility testing

| Drug | Candida species | CLSI M60 clinical breakpoints^1^ µg/mL | | | | ECV (CLSI M59)^2^  µg/mL | |
| --- | --- | --- | --- | --- | --- | --- | --- |
|  |  | S | I | SDD | R | WT | Non WT |
| Amphotericin B | *C. albicans, C. parapsilosis, C. tropicalis, C. glabrata, C. krusei* |  |  |  |  | ≤2 | >2 |
| Fluconazole | *C. albicans, C. parapsilosis, C. tropicalis* | ≤2 |  | 4 | ≥8 |  |  |
|  | *C. glabrata* |  |  | ≤32 | ≥64 |  |  |
|  | *C. krusei* |  |  |  |  |  |  |
| Voriconazole | *C. albicans, C. parapsilosis, C. tropicalis* | ≤0.2 | 0.25-0.5 |  | ≥1 |  |  |
|  | *C. glabrata*^a^ |  |  |  |  | ≤0.5 | >0.5 |
|  | *C. krusei* | ≤0.5 | 1 |  | ≥2 |  |  |
| Caspofungin | *C. albicans, C. tropicalis, C. krusei* | ≤0.25 | 0.5 |  | ≥1 |  |  |
|  | *C. parapsilosis* | ≤2 | 4 |  | ≥8 |  |  |
|  | *C. glabrata* | ≤0.12 | 0.25 |  | ≥0.5 |  |  |

CLSI, Clinical and Laboratory Standards Institute; ECV, Epidemiological Cutoff Value; S, susceptible; I, intermediate; SDD, Susceptible Dose Dependent; R, resistant; WT, wild-type.

^a^ECV for voriconazole is not available in CLSI M59 document therefore adopted from the 2011 proposed ECV-based criteria^3^

Reference:

1. M60: Performance Standards for Antifungal Susceptibility Testing of Yeasts, 1st Edition. Available at: <https://clsi.org/standards/products/microbiology/documents/m60/>
2. M59Ed2 | Epidemiological Cutoff Values for Antifungal Susceptibility Testing. Available at: <https://clsi.org/standards/products/microbiology/documents/m59/>
3. Pfaller, M. A. *et al.* Clinical breakpoints for the echinocandins and Candida revisited: integration of molecular, clinical, and microbiological data to arrive at species-specific interpretive criteria. *Drug Resist. Updat. Rev. Comment. Antimicrob. Anticancer Chemother.* **14**, 164–176 (2011)
